# Astroglial Inhibition of NF-κB Does Not Ameliorate Disease Onset and Progression in a Mouse Model for Amyotrophic Lateral Sclerosis (ALS)

**DOI:** 10.1371/journal.pone.0017187

**Published:** 2011-03-18

**Authors:** Claudia Crosio, Cristiana Valle, Arianna Casciati, Ciro Iaccarino, Maria Teresa Carrì

**Affiliations:** 1 Department of Physiological, Biochemical and Cell Science, University of Sassari, Sassari, Italy; 2 Fondazione Santa Lucia IRCCS, c/o CERC, Rome, Italy; 3 Cell Biology and Neurobiology Institute, National Research Council, Monterotondo Scalo, Italy; 4 Department of Biology, University of Rome “Tor Vergata”, Rome, Italy; National Institutes of Health, United States of America

## Abstract

Motor neuron death in amyotrophic lateral sclerosis (ALS) is considered a “non-cell autonomous” process, with astrocytes playing a critical role in disease progression. Glial cells are activated early in transgenic mice expressing mutant SOD1, suggesting that neuroinflammation has a relevant role in the cascade of events that trigger the death of motor neurons. An inflammatory cascade including COX2 expression, secretion of cytokines and release of NO from astrocytes may descend from activation of a NF-**κ**B-mediated pathway observed in astrocytes from ALS patients and in experimental models. We have attempted rescue of transgenic mutant SOD1 mice through the inhibition of the NF-κB pathway selectively in astrocytes. Here we show that despite efficient inhibition of this major pathway, double transgenic mice expressing the mutant SOD1^G93A^ ubiquitously and the dominant negative form of I**κ**Bα (I**κ**BαAA) in astrocytes under control of the GFAP promoter show no benefit in terms of onset and progression of disease. Our data indicate that motor neuron death in ALS cannot be prevented by inhibition of a single inflammatory pathway because alternative pathways are activated in the presence of a persistent toxic stimulus.

## Introduction

Amyotrophic Lateral Sclerosis (ALS), the most common adult-onset motor neuron disease, is usually fatal within five years of onset and is characterized by the degeneration of upper and lower motor neurons. Most ALS cases are sporadic, but about 5–10% of patients inherit the disease, typically in an autosomal dominant manner (familial ALS, FALS). Family-based linkage studies have led to the identification of twelve loci and eight genes for FALS, as well as three loci for ALS with frontotemporal dementia [1]. Approximately 20% of familial cases are caused by mutations in the gene coding for Cu/Zn superoxide dismutase (SOD1), and following linkage studies published in 1993, many different transgenic animal and cellular models of human SOD1 mutations have been developed, increasing our knowledge about the pathogenesis of both sporadic and familial forms of ALS [2].

Current hypotheses for the biology underlying sporadic and familial ALS forms in humans represent non-competing mechanisms that are likely to converge in various unfortunate patterns to mediate selective motor neuron degeneration [3]. Mutant SOD1 toxicity has been linked to oxidative damage, accumulation of intracellular aggregates, mitochondrial dysfunction, defects in axonal transport, growth factor deficiency, glial cell pathology, and glutamate excitotoxicity. A growing body of evidence indicates that non-neuronal cells contribute to the disease process in animal [4], [5], [6], [7], [8] and cellular [4], [9], [10] models overexpressing mutant SOD1. As a consequence, motor neuron death in ALS is considered as a “non-cell autonomous” process, with astrocytes playing a critical role in disease progression [11]. Astrocytes have many functions relevant to motor neuron physiology. First, they express the most important glutamate transporter EAAT2/GLT-1, thus contributing to the clearance of this neurotransmitter; deficiency of astroglial EAAT2/GLT-1 causes severe motor neuron loss [12] and alteration of this transporter has been repeatedly invoked as a cause contributing to ALS [3]. Second, astrocytes are the major source of both trophic [13] and toxic factors [4] for motor neurons. Several cytokines have been proposed to play a role in ALS as reinforcing signals from glia cells, including interleukin-6 (IL6), tumour necrosis factor α (TNFα), monocyte chemoattractant protein-1, monocyte colony-stimulating factor (MCSF) and transforming growth factor β1 (TGFβ1) that were found increased in cerebrospinal fluid, plasma and epidermis from ALS patients, although with sometimes conflicting results [14]. In addition, the production of nitric oxide and the activation of cyclooxygenase type 2 (COX2) aggravate the toxic effects of mutant SOD1 in several experimental models for ALS. The production of all those proinflammatory mediators may be secondary to the induction of the transcription factor NF-**κ**B, which is activated in the presence of reactive oxygen species (ROS) and by many other different signalling molecules associated with ALS onset and progression [15], [16]. NF-**κ**B activation has been observed in astrocytes from ALS patients and in human cells expressing mutant SOD1 [17]. NF-**κ**B also regulates the expression of COX2 that may cause an increase in the synthesis of prostaglandins, which trigger astrocytic glutamate release and induce free radical formation, thus contributing to both excitotoxicity and oxidative damage. Indeed, treatment with COX2 inhibitors markedly protects motor neurones and significantly prolongs survival of ALS mice [18].

An approach that has been widely used to study cell specific NF-**κ**B function in mice is to inhibit its activation by the (over)expression of various degradation-resistant mutant isoforms of I**κ**Bα, the physiological inhibitor of NF-**κ**B. These proteins, that may be collectively termed I**κ**B-DR (I**κ**B-degradation resistant, [19], act in a dominant negative manner to block NF-**κ**B activation, by impairing its nuclear translocation and transcriptional activation [20]. To address the contribution of astroglial NF-**κ**B and, more generally, the contribution of astrocytosis to ALS onset and progression, we generated a mouse line expressing an I**κ**Bα-DR (I**κ**BαAA) in astrocytes only, under control of the astrocyte-specific glial fibrillary acidic protein (GFAP) promoter, and crossbred them with transgenic mice over-expressing ALS-typical mutant SOD1^G93A^. We demonstrate that GFAP-I**κ**BαAA transgenic mice grow normally, although they are highly sensitive to lipopolysaccharides (LPS)-induced toxaemia. However, SOD1^G93A^ mice made deficient for NF-**κ**B activation in astrocytes do not show an ameliorated ALS phenotype despite a slight but significant reduction in astrogliosis at onset.

## Results

### Generation of IκBαAA transgenic mice and phenotypical characterization

**Figure 1:**
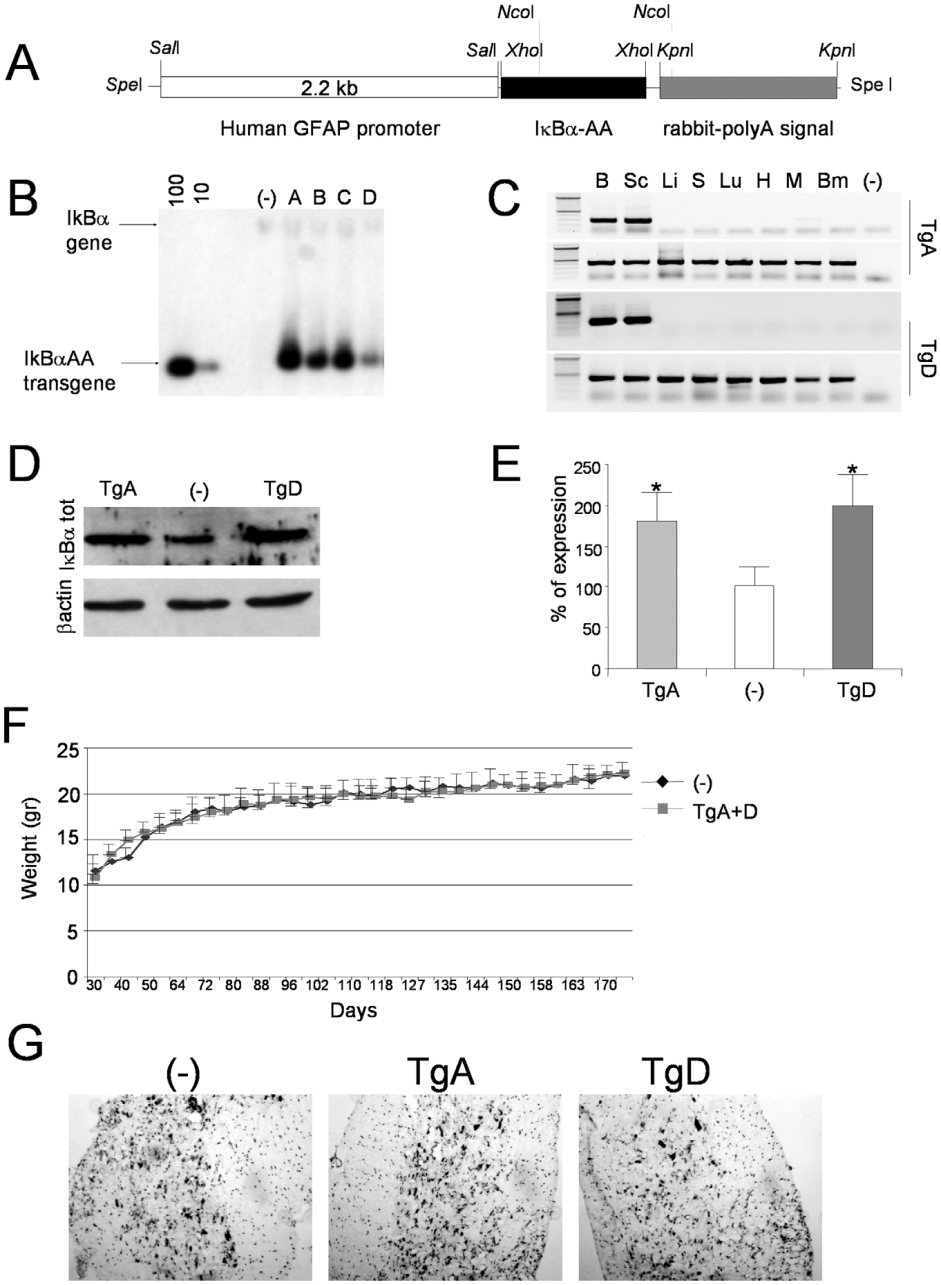
Characterization of GFAP-IκBαAA transgenic mice lines. (A) Schematic representation of transgene construction. (B) Southern blot analysis performed on 10 µg of genomic DNA extracted from the indicated transgenic lines (Tg-A, -B, -C and -D) and non transgenic mice (-) digested with *Xho*I. Standards to determine copy numbers were obtained according to [58]. Fragments corresponding to IκBα gene (∼14 Kbp) and transgene (950 bp) are indicated with arrows. (C) RT-PCR analysis of GFAP-I**κ**BαAA mRNA expression in different tissues of two transgenic lines (Tg-A and Tg-D); brain (B), spinal cord (Sc), liver (Li), spleen (S), lung (Lu), heart (H), muscle (M) and bone marrow (Bm); as negative control we amplified RNA from Sc of TgA mice (-). To selectively amplify transgene, reverse primer was designed on rabbit-polyA signal. GAPDH cDNA was amplified as control. (D) Western blot analysis on spinal cord protein extract from transgenic lines (Tg-A and Tg-D) using antibodies against IκBα and β-actin. Non transgenic mice (-) were used as control. (E) Densitometric quantification of results obtained in (D). These results are the means +/− SEM of three independent experiments performed with two mice for each genotype. *P<0,05, compared to the corresponding non Tg (-) mice. (F) The expression of the transgene GFAP-I**κ**BαAA has no effect on growth of mice. Results are expressed as the mean of body weight recordered twice weekly from 30 to 170 days of age +/− SEM. N = 22 mice/group (10 males and 12 females). (G) Cresyl -violet staining on spinal cord cryosections from 15 weeks old transgenic mice (TgA and TgD) and non transgenic (-) mice.

We generated tissue-restricted NF-**κ**B knockout mice through targeting of a dominant-negative form of I**κ**Bα in glial cells. When the two N-terminal serines of I**κ**Bα have been mutated into alanines I**κ**Bα-S32/36A, I**κ**BαAA), the protein cannot be phosphorylated and degraded and therefore acts as a super-repressor of NF-**κ**B activity. In its presence, every NF-**κ**B/Rel complex is sequestered in the cytoplasm, prevented from DNA binding and consequently from activating transcription [21]. We have generated transgenic mice over-expressing the dominant negative form of I**κ**Bα selectively in astrocytes (GFAP-I**κ**BαAA), using the human GFAP promoter [22]. As shown in Figure 1, we have generated four different independent transgenic lines, indicated from A to D, that have integrated in their genome a number of transgene copies included between 10–20 and 200 as suggested by band intensity comparison (Figure 1B).

Transgene expression was analyzed by RT-PCR on total RNA extracted from different tissues of mice generated from founder A and D (indicated as TgA and TgD, respectively). As shown in Figure 1C, different levels of transgene expression were obtained, but in all cases expression of GFAP-I**κ**BαAA was predominant in neuronal tissue, with no ectopic expression in peripheral tissues, except faintly in muscle of transgenic line A. Total proteins, extracted from spinal cord samples from TgA, TgD and non-transgenic mice (-) were tested by Western blot for I**κ**Bα expression. As shown in Figure 1D–E, I**κ**Bα overexpression is clearly detectable and significantly increased in both transgenic lines in tissue samples in which astrocytes are not the only cellular population. Expression of I**κ**Bα-AA in both transgenic lines does not induce any obvious phenotype as confirmed by their normal growth of mice (Figure 1F). Standard cresyl violet histopathology did not reveal any gross morphological anomalies in the architecture of the naive spinal cord in GFAP- I**κ**BαAA-A/D mice (figure 1G).

**Figure 2:**
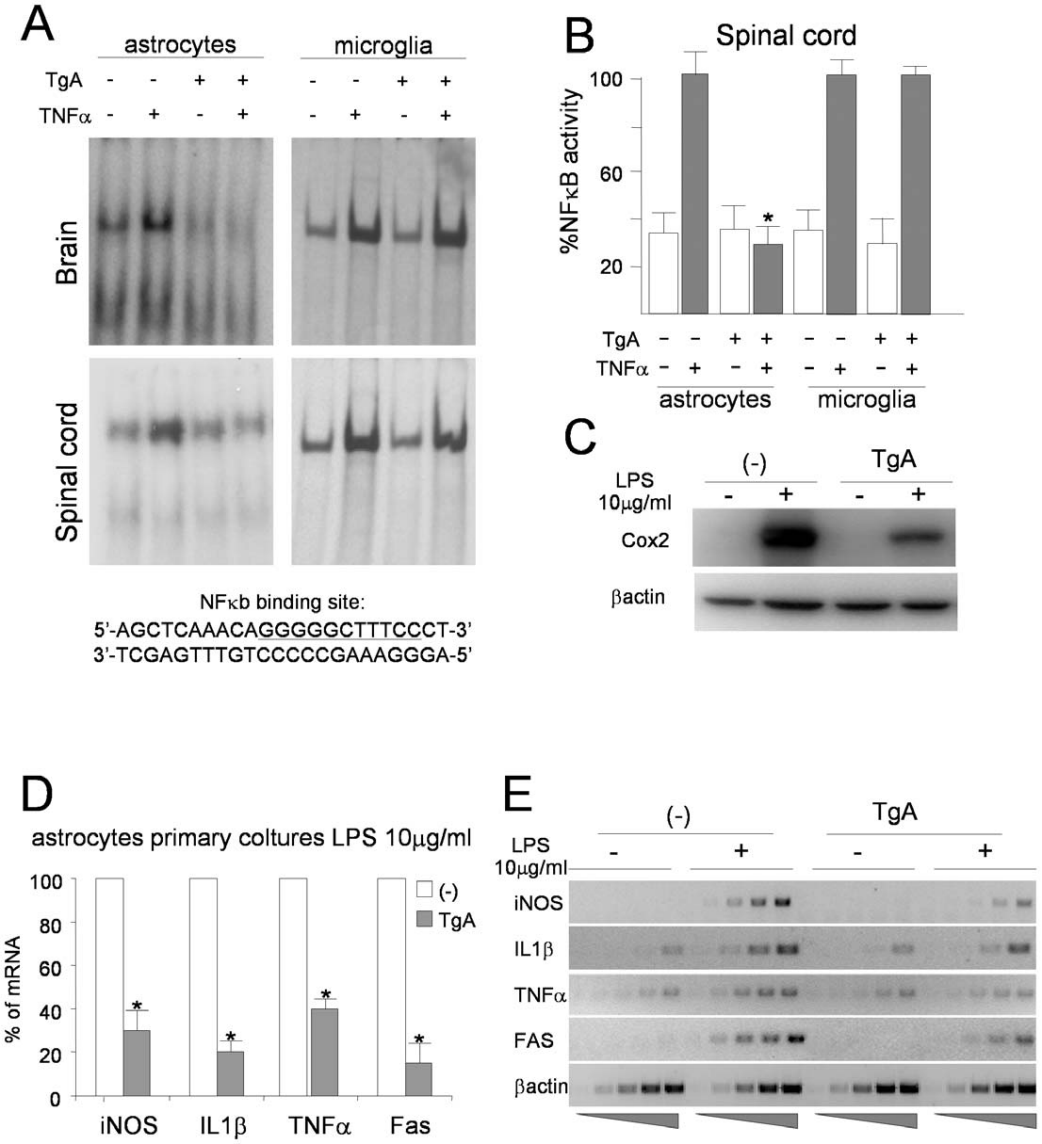
Inhibition of NF-κB activity in astrocytes from GFAP-IκBαAA mice. (A) EMSA of nuclear protein extracts (5 µg) from primary astrocytes and microglia from brain and spinal cord of transgenic mice line A (Tg-A) and non transgenic mice (-) stimulated or unstimulated for 20 min with recombinant TNFα (10 ng/ml) before analysis. For NF-**κ**B consensus probe see underlined sequence below. (B) Quantification of the experiments in (A) performed with spinal cord astrocytes and microglia by densitometric assay. Results obtained from non transgenic stimulated cells were defined as 100%. Results are expressed as the means +/− SEM of three independent experiments. *P<0,01. Effect of 3 h LPS treatment (10 µg/ml) on different NF-**κ**B controlled gene in primary astrocytes from non transgenic (-) or GFAP-I**κ**BαAA (TgA) transgenic mice. (C) Western blot analysis on protein extract using antibodies against COX2 and β-actin. (D) semi-quantitave PCR for iNOS, IL-1β, TNFα, Fas mRNA expression. Results obtained with cells from stimulated non transgenic mice are defined as 100% and compared with their corresponding results from stimulated transgenic samples. Data are normalized to β-actin. *p<0.05 **p<0.01. Results represent the mean +/−SEM of three groups. (E) Representative semi-quantitave PCR performed on cDNA from total RNA extracted from astrocytes primary cultures from non transgenic (-) and transgenic (Tg-A) mice untreated and treated with LPS 10 µg/ml.

To confirm the ability of I**κ**BαAA to prevent NF-**κ**B activation, electrophoretic mobility shift assay (EMSA) was performed on nuclear extracts from astrocytes isolated from brain (figure 2A, upper panels) and spinal cord (figure 2A, lower panels) of non-transgenic (-) and GFAP-I**κ**BαAA transgenic mice (Tg-A) (Figure 2A). Astrocytes from control mice stimulated with TNFα exhibited a significant induction of NF-**κ**B DNA binding activity, whereas no induction was detected in astrocytes from transgenic mice (Figure 2A, left panels). To confirm the cell specificity of transgene expression, similar EMSA experiments were performed on microglial cells isolated from brain and spinal cord of GFAP-I**κ**BαAA mice and non-transgenic littermates (Fig. 2A, right panels). As expected, when stimulated with TNFα, GFAP- I**κ**Bα-AA microglial primary cultures showed an increase in NF-**κ**B DNA binding activity comparable to non-transgenic microglial cells, demonstrating the complete functionality of the NF-**κ**B pathway in this cell type. Quantification of results is shown in Figure 2B. Similar results were obtained with line TgD in primary cultures (data not shown). At molecular level, the impairment of NF-**κ** signalling in primary astrocytes of GFAP-I**κ**BαAA transgenic mice causes a severe reduction in the expression of many different **κ**B controlled genes upon challenge with lipopolysaccharide (LPS). Cox2 levels were assayed at protein level (figure 2C), while iNOS, IL-1β, TNFα, FAS at mRNA level (figure 2D and 2).

### NF-κB inhibition in glial cells does not influence onset and progression of ALS, caused by SOD 1 mutations

To assess the contribution of NF-**κ**B inhibition in glial cells in the pathogenesis of ALS, we have generated two different double transgenic mice lines by crossing SOD1^G93A^ mice with the two independent GFAP-I**κ**BαAA transgenic lines described above, in order to exclude a founder effect. The effect of NF-**κ**B deficiency in glial cells on severity of motor neuron disease was examined using cohorts of GFAP-I**κ**BαAA (indicated as Tg), SOD1^G93A^ (indicated as G93A) and GFAP-I**κ**BαAA/SOD1^G93A^ (indicated as double Tg) transgenic mice.

**Figure 3:**
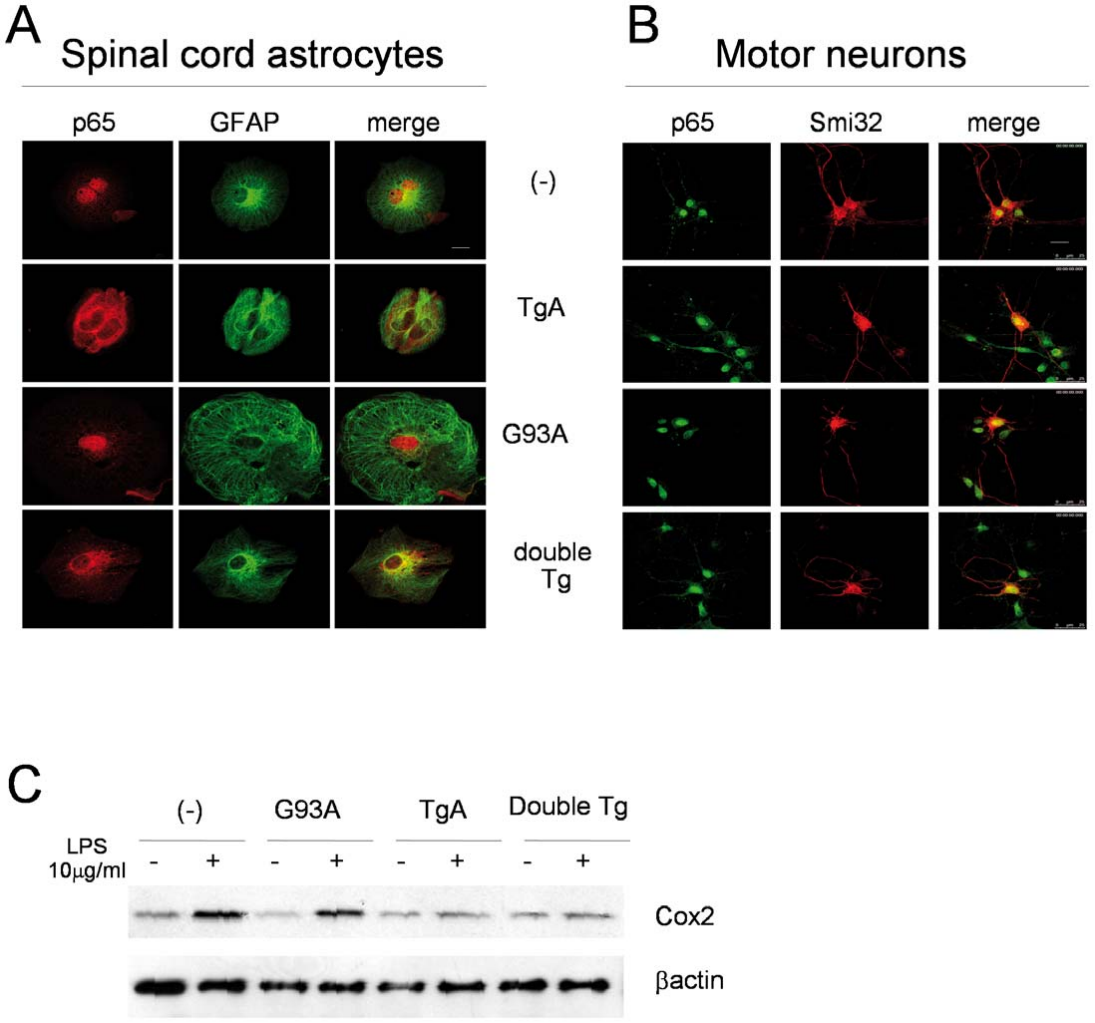
Inhibition of p65 nuclear translocation in spinal cord primary cultures of astrocytes from GFAP-IκBαAA and double transgenic GFAP-IκBαAA/SOD1^G93A^ mice after treatment with LPS (10 µg/ml for 20 min). (A) Immunofluorescent labeling of spinal cord primary astrocytes from non transgenic (-), transgenic GFAP-I**κ**BαAA (TgA), SOD1^G93A^ (G93A) and double transgenic GFAP-I**κ**BαAA/SOD1^G93A^ (double Tg) mice using antibodies against p65 and GFAP. Scale bar 20 µm. (B) Immunofluorescent labeling of primary motorneurons from mice as in A using antibodies against p65 and SMI32. Scale bar 20 µm; (C) Western Blot analysis of protein extracts for spinal cord primary astrocytes from mice as in (A) using antibodies against COX2 and β-actin, after stimulation with LPS 10 µg/ml for 16 h.

We initially confirmed cell-specific inhibition of NF-**κ**B by stimulating primary cultures of motor neurons, microglial (data not shown) and glial cells from transgenic (GFAP-I**κ**BαAA, indicated as TgA and SOD1^G93A^, indicated as G93A), double transgenic (SOD1^G93A^/GFAP-I**κ**BαAA, indicated as double Tg) and non-transgenic littermates (-) with LPS (or TNFα data not shown) for 20 min and determining NF-**κ**B (p65) nuclear translocation by immunofluorescence. As expected, we did not observe NF-**κ**B nuclear translocation in glial cells isolated from spinal cord (Figure 3A) of mice expressing I**κ**BαAA, independently from SOD1^G93A^ presence (TgA and double Tg) in contrast of motor neurons of all genotypes (Figure 3B). Notably, despite the observation that mutant SOD1 stably expressed in a neuronal cell line may induce up-regulation of NF-**κ**B [17], in primary astrocytic cultures derived from double transgenic mice the super-repressor I**κ**BαAA is still able to prevent p65 nuclear translocation upon LPS (or TNFα data not shown) stimulation. The impairment of NF-**κ**B signalling in double transgenic mice was confirmed by the lack of Cox2 induction upon LPS stimulation, as in TgA mice (figure 3C).

**Figure 4:**
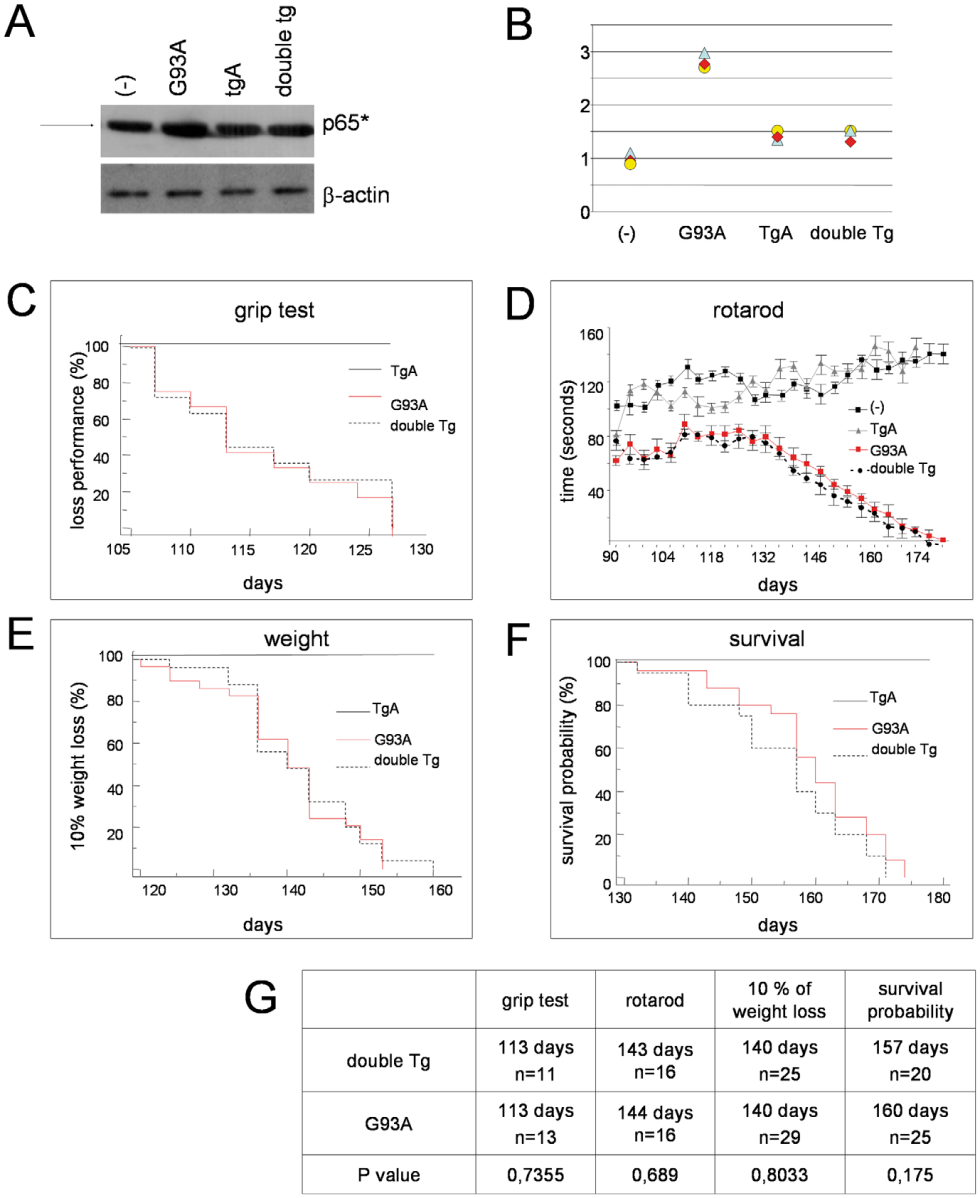
Selective expression of IκBα-AA in astrocytes does not ameliorate ALS onset or progression. (A) NF-**κ**B activation in spinal cord extracts of non transgenic (-), transgenic GFAP-I**κ**BαAA (TgA), SOD1^G93A^ (G93A) and double transgenic GFAP-I**κ**BαAA/SOD1^G93A^ (double Tg) mice. NF-**κ**B activation was evaluated by Western blot analysis with an antibody that specifically recognizes the activated form of p65 (p65*). The arrowhead indicates p65* migrating at exactly 65 kD. 15 µg of proteins/sample were loaded, and blots were probed for β-actin as a control. (B) Schematic representation of result obtained in (A). Selective expression of I**κ**Bα-AA in astrocytes does not ameliorate grip strength (C), rotarod performance (D), weight decline (E) and survival (F) in double transgenic mice GFAP-I**κ**BαAA/SOD1^G93A^. (G) Median values, sample size (males∶ female ratio was always 1∶1) and p values for the above experiments are reported.

Alteration of NF-**κ**B-signalling has been widely linked to ALS onset and progression, [15], [17], [23], [24]. Using an antibody that specifically recognizes an epitope overlapping the nuclear localization signal of the p65 subunit and, thus, selectively binding to the activated form of p65 (indicated as p65*), we evaluated the activation of NF-**κ**B in single and double transgenic mice at the onset stage by Western blot. As expected, we observed in G93A mice an increased NF-**κ**B activation, that is prevented in double transgenic mice (figure 4A and 4B). These data indicate the effectiveness of GFAP-I**κ**BαAA transgenic mice in inhibiting NF-**κ**B signalling in the astrocytes of G93A spinal cord.

Nonetheless, as shown in Figure 4, the impairment of NF-**κ**B signalling in glial cells did not influence the rate of mortality in the double transgenic mice lines. SOD1^G93A^ and double transgenic mice developed ALS in a comparable manner (data reported in Figure 4 refer only to the double transgenic line generated by crossing GFAP-I**κ**BαAA line A with SOD1^G93A^, but they are indistinguishable from those obtained with the other double transgenic line). There was no difference in disease onset measured either evaluating motor performance in grip test (Figure 4C) and rotaroad (Figure 4D) or by 10% of weight loss (Figure 4E). Disease progression was also similar and the median lifespan was 160 and 157 days for SOD1^G93A^ and double transgenic mice, respectively (figure 4F). Notably, the life span we observed in our transgenic lines is similar to the one observed in the original B6.Cg-Tg(SOD1-G93A)1Gur line (from The Jackson Laboratory, 50% survive at 157.1+/−9.3 days, 99,99% B57BL/6J, http://jaxmice.jax.org/strain/004435.html).

### Selective expression of IκBαAA in astrocytes causes a delayed microgliosis and astrocytosis at the onset of disease only

**Figure 5:**
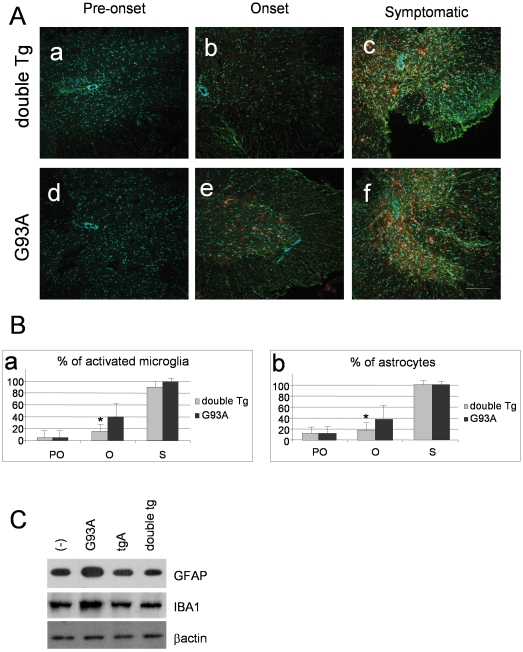
Selective expression of IκBαAA in astrocytes causes a weakly delayed microgliosis and astrocytosis only at the onset of disease (120 days). (A) Immunofluorescence against GFAP (green) and Cd11b (red) in sections of lumbar spinal cord from 100 days (pre-onset), 120 days (onset) and 140 days (symptomatic) old double transgenic mice GFAP-I**κ**BαAA/SOD1^G93A^ (a,b,c) and SOD1^G93A^ mice (d,e,f). Scale bar 100 µm. (B). Schematic representation of counting results in (A). Data are expressed as percentage (mean+/−SEM) of activated microglia (a) or astrocytes (b) from 3 different animals from the three different disease stages: pre-onset (PO); onset (O); symptomatic (S). The number of cells recorded from SOD1^G93A^ mice at the symptomatic stage was considered 100%. *p<0.05. (C) GFAP and IBA1 protein levels were evaluated by Western blot on 20 µg of proteins/sample. Blots were probed for β-actin as a control.

**Figure 6:**
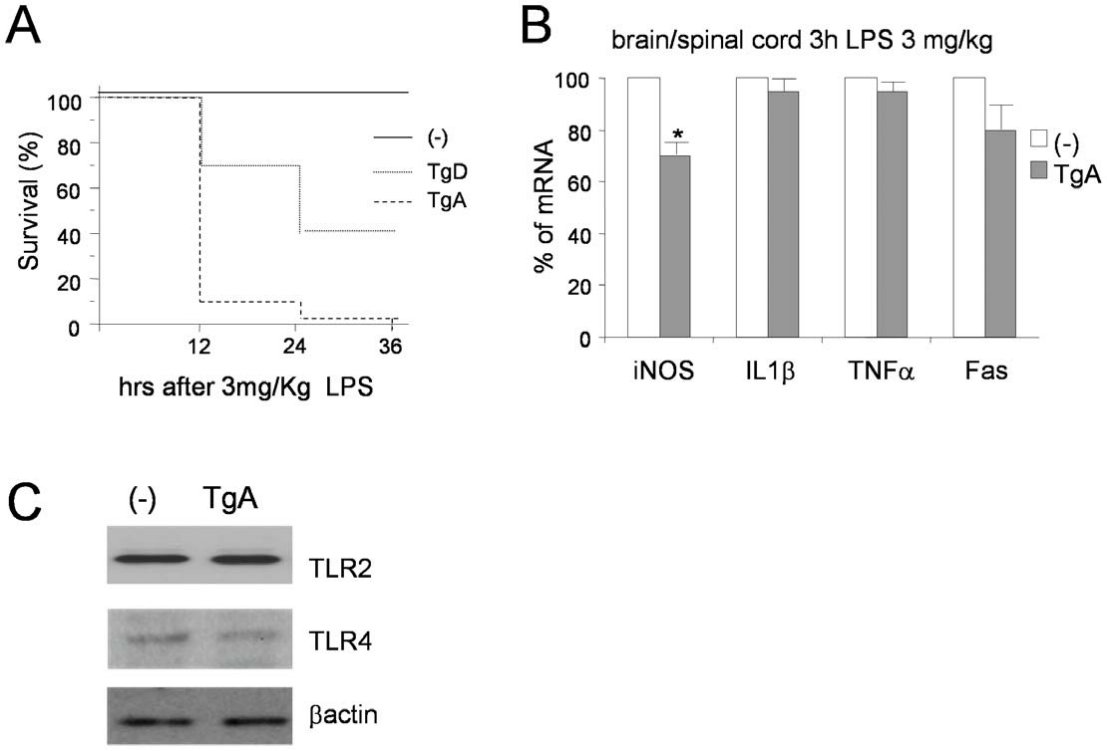
Effects of LPS stimulation in GFAP-IκBαAA and GFAP-IκBαAA/SOD1^G93A^ mice. (A) Effect of intraperitoneally LPS injection on survival of 10 weeks old non transgenic (-) and transgenic (Tg-A and Tg-D) mice. N = 20 mice/group (10 males and 10 females). (B) Effect of 3 h LPS treatment on iNOS, IL-1β, TNFα, Fas mRNA expression in tissues as brain or spinal cord from GFAP-IκBα-AA mice as determined by semi-quantitave PCR. Results obtained with tissues from stimulated non transgenic mice are defined as 100% and compared with their corresponding results from stimulated transgenic samples. Data are normalized to β-actin. *p<0.05 **p<0.01. Results represent the mean +/−SEM of three animals/group. (C) Western blot analysis on spinal cord protein extract from non transgenic (-) and GFAP-I**κ**BαAA transgenic mice (TgA) using antibodies against TRL2, TRL4 and β-actin.

Astrocytosis and microgliosis are non-neuronal events that likely contribute to the neurodegenerative process in ALS. NF-**κ**B is a transcription factor involved in gene expression of many different inflammatory molecules whose function is crucial for microglial activation and induction of reactive astrogliosis. We therefore investigated whether glial NF-**κ**B deficiency in mutant SOD1 mice had any effect on the expression of CD11b, a marker of microglial activation, and GFAP, a marker of astrogliosis. Immunoreactivity was assessed in the spinal cord of normal mice, SOD1^G93A^ and double transgenic mice at pre-onset (100 days), onset (120 days) and symptomatic phase (140 days) of disease progression. As shown in Figure 6, we observed a slight, but statistically significant, reduction of both CD11b and GFAP positive cells at the onset stage in double transgenic mice with respect to SOD1^G93A^ mice. This effect was lost at later stages of disease and the astroglial inhibition of NF-**κ**B in the SOD1^G93A^ background was not sufficient to reduce neuroinflammation in the spinal cord of double transgenic mice. Western blot analysis confirms the reduction of GFAP reactive protein and IBA1, a marker of microglial activation, in spinal cord extract from double Tg mice with respect to G93A mice (figure 5C).

### Astroglial inhibition of NF-κB is deleterious upon LPS-induced septic shock

The data presented above indicate that astroglial inhibition of NF-**κ**B does not affect motor neuron survival in an ALS genetic model, although astrocyte dysfunction, via a number of pathways including the NF-**κ**B one, has been invoked as a potential mediator of disease progression. Previous studies have demonstrated that a chronic stimulation of innate immunity, via sub-lethal systemic injection of LPS, can exacerbate ALS disease progression [25], [26]. Thus, we decided to investigate the effect of LPS on G93A/GFAP-I**κ**BαAA double transgenic mice to evaluate whether the NF-**κ**B impairment in astrocytes may have any effect on LPS exacerbation of ALS disease progression. This experiment revealed unfeasible since, surprisingly, when 10-week-old GFAP-I**κ**BαAA single transgenic mice were challenged with a sub-lethal dose of LPS (1, 3 and 5 mg/kg, Sigma-Aldrich, from *E. coli* strain 111:B4), at all doses tested we observed a premature death of mice from both GFAP-I**κ**BαAA transgenic lines (Figure 6A).

At the molecular level, we observed that 3 h hours after LPS treatment, a significant reduction of iNOS induction occurred in the spinal cord of TgA (figure 6B). IL1β, TNFα and Fas showed a modest reduction in spinal cord from TgA mice, that does not reach statistical significance (figure 6B).

In the attempt of investigating the hypersensitivity of GFAP-I**κ**BαAA transgenic mice to LPS, we have analyzed the expression level of the toll-like receptors 2 and 4, which have been indicated as LPS mediators [27], [28], [29]. As shown in figure 6C, TRL2 and TRL4 levels are equal in the spinal cord from TgA compared to non transgenic mice.

## Discussion

In the past five years, neuroinflammation has been recognized as a key event in disease onset and progression, modulating death of motor neurons in ALS. Yet, several lines of evidence indicate that gliosis may actually exert very different effects in the diseased spinal cord as it may mediate either beneficial or harmful events. Indeed, activated astrocytes can mediate neuroprotection by preserving motor neuron survival through the release of anti-inflammatory cytokines, neurotrophins, and growth factors [30]. On the other hand, reactive astrocytes can participate directly in inflammatory reactions expressing inflammation markers including iNOS and COX2 and other mediators including prostaglandins, IL-6, TNFα, IL-1β and NGF [30].

As demonstrated by two independent groups, astrocytes may be the primary cell types where mutant SOD1 exert its toxic effects on motor neurons by releasing some not yet identified molecule(s) [9], [10]. Moreover, genetic evidence obtained in mice models for ALS indicate that lack of expression of mutant SOD1 in GFAP expressing astrocytes [7], but not astrocyte ablation [31], sharply slowed disease progression. These findings demonstrate that while astrocyte signalling is an important factor in the aetiology of motor neuron diseases, astrocyte proliferation itself does not play a significant role.

A number of different signalling molecules released by astrocytes are likely to be under the control of the transcription factor NF-**κ**B, a key molecule responding to both redox and inflammatory stimuli [20]. In order to explore the contribution of NF-**κ**B regulated gene expression in astrocytes to ALS onset and progression, we developed transgenic mice expressing a super-repressor of NF-**κ**B specifically in GFAP-positive astrocytes (GFAP-I**κ**BαAA). This type of cell-specific repression has been widely used to efficiently block NF-**κ**B activation, because the degradation-resistant I**κ**B mutant interferes preferentially with the activity of canonical NF-**κ**B dimers [19], [32].

Using a similar approach, it has been demonstrated that inactivation of NF-**κ**B activity in astrocytes can either protect neurons from different insults (improving functional recovery following spinal cord injury [33], [34] and experimental autoimmune encephalomyelitis [35], and promoting survival of retinal neurons following ischemic injury [36]), or have no effect on preventing neuronal death in cerebral ischemia [37]. The two transgenic GFAP-I**κ**BαAA mouse strains used in this study grow normally and they display impairment in NF-**κ**B activation upon LPS or TNFα stimulation specifically in astrocytes (Figures 2, 3).

Unexpectedly, we report here that NF-**κ**B downregulation in astrocytes fails to influence onset, severity, or progression of disease in a mutant SOD1-based ALS mice model. Though we observed a slight but significant reduction in the percentage of activated microglia and astrocytes at onset stage, neither survival nor motor performance of double transgenic mice differ from those of single transgenic ALS mice (Figure 4–5). We may speculate that the reduction observed in GFAP markers in double transgenic mice with respect to G93A mice at the onset of the disease is due to a delay in astrogliosis for different reasons: i) we observe a similar reduction in CD11b positive cells, that do not express the transgene (figure 2); ii) astroglial and neuronal NF-**κ**B has been shown not to be a critical regulator of survival under non pathological conditions [19], [33]; iii) at end stage of disease the level of GFAP positive cells in double transgenic mice is comparable to the G93A transgenic mice.

Diminished expression of mutant SOD1 in astrocytes delays microglial activation and significantly reduces disease progression in ALS mice [38], [39], a fact suggesting that noxious signals from astrocytes do contribute significantly to the progression of non-cell autonomous killing of motor neurons in ALS via activation of both microglia and astrocytes, although ablation of proliferating microglia or astrocytes does not affect motor neuron degeneration in SOD1-ALS [31], [40]. Here we show that the inhibition of NF-**κ**B signalling in astrocytes does not affect motor neuron survival in a SOD1-linked ALS mouse model. This result indicates that the toxic effects generated by expression mutant SOD1 in astrocytes are independent from NF-**κ**B signalling. Other pathways can be activated in astrocytes that parallel or compensate the NF-**κ**B inhibition leading to the same inflammatory response at late stage of the disease in both G93A and G93A/GFAP-I**κ**BαAA transgenic mice.

Although inhibition of NF-**κ**B in astrocytes does not affect motor neuron death upon expression of mutant SOD1, it is crucial in experiments of LPS-induced toxaemia (Figure 6). In fact in our GFAP-I**κ**BαAA transgenic mice relatively low doses of LPS evoke a striking reduction in survival, roughly dependent on transgene copy number. Our results are partially in contrast with those obtained in another GFAP-I**κ**Bα-AA transgenic line, which responds to LPS treatment as non-transgenic mice [41]. This discrepancy can at least partially be explained by the slightly different transgenic construction and considering that individual transgenic mouse strains over-expressing I**κ**Bα-DR proteins in a given tissue often show phenotypes of varying severity in relation to the extent of NF-**κ**B inhibition achieved. Although we did not investigate extensively the molecular mechanism underlying the premature death of GFAP-I**κ**BαAA mouse after LPS systemic injection, a significant reduction in mRNAs levels of NF-**κ**B controlled genes iNOS, IL-1β, TNFα and Fas occurs after LPS treatment in primary astrocytes and to a lower extent in spinal cord and brain of transgene mice (figure 2 and 6). Moreover the hypersensitivity of GFAP-I**κ**BαAA mouse to LPS does not seem to be linked to toll-like receptors expression (figure 6). Most importantly, our findings parallel the results obtained in other transgenic mice lines in which the inhibition of NF-**κ**B activation was restricted to endothelial cells [42] or in hepatocytes [43]. Mice expressing I**κ**BαAA, under the control of the *Tie* endothelial promoter, showed a marked increase in vascular permeability and rapidly died within 60 hours after LPS challenge [42]. Considering that, in healthy neural tissue, astrocytes play critical roles in many functions, including blood-brain barrier permeability, regulation of blood flow, homeostasis of extracellular fluid, ions and transmitters, energy provision, and regulation of synapse function and synaptic remodelling [44], the disturbance or loss of these functions has the potential to underlie LPS hypersensitivity. Many lines of evidence, in fact, indicate that diffusible inflammatory molecules produced by astrocytes can prevent activation of microglia and macrophages and might lessen some symptoms of neural inflammation [44], [45]. Moreover, loss or attenuation of reactive astrocyte functions might worsen outcome after various kinds of CNS insults, e.g. through excitotoxic failure of glutamate uptake [46], [47] or increased inflammation or infection due to loss of astrocyte barrier functions [46], [48], [49]. Indeed, a possible explanation of our results could lie in the dysfunction of the blood-brain barrier, in which astrocytes are localized, with a consequent alteration of the transport of prostaglandin, the final signal transduction mediators from the periphery to the brain during fever response [50], [51]. Given the dual role of reactive astrocytes, in our opinion it is not surprising that similar astrocityc inhibition of NF-**κ**B in mice may produce different outcomes (neurotoxicity versus neuroprotection) upon different experimental insults [33], [35].

In conclusion, in this study, we have used two independent lines of transgenic GFAP-I**κ**BαAA mouse strains, obtaining overlapping results in relation to SOD1^G93A^ induced toxicity despite the fact that TgA mice show a slight expression of the transgene in muscle, but not in other tissues checked. We conclude that the lack of phenotype is not due to a founder effect and/or to (minimal) transgene mis-expression.

On the other hand, though alteration of NF-**κ**B-signalling has been widely linked to ALS onset and progression, [15], [17], [23], [24], our results suggest that, upon chronic inhibition of the NF-**κ**kB pathway specifically in astrocytes, compensatory effects occur in double transgenic mice where the toxic activity of mutant SOD1 is still present. A similar effect was observed by Gowing and coll [52]: genetic ablation of TNFα (TNFα gene knock out) does not affect motor neuron disease caused by SOD1 mutations, although higher level of TNFα and of its soluble receptors have been shown in plasma from ALS patients [53], [54]. This indicates that the simple interference with a single aspect of the neuroinflammatory response is not *per se* sufficient to modify ALS onset and progression induced by SOD1 mutations and further supports the concept that it is the convergence of damage developed within multiple cell types, including neighbouring non-neuronal cells, which is crucial to neuronal dysfunction [39].

This may at least partially explain why several anti-inflammatory compounds that have been tested in phase II/III clinical trials in ALS during the last 15 years [55] have provided no beneficial effect on ALS patients and support the need of a multi-drug, synergistic therapy in ALS, as suggested from studies in mice [56].

## Materials and Methods

### Ethics Statement

All animal procedures have been performed according to the European Guidelines for the use of animals in research (86/609/CEE) and the requirements of Italian laws (D.L. 116/92). The ethical procedure has been approved by the Animal welfare office, Dept. of Public Health and Veterinary, Nutrition and Food Safety, General Management of Animal Care and Veterinary Drugs of the Italian Ministry of Health (Application number 32/08 of July 7, 2008; Approval number 744 of January 9, 2009).

At the indicated time, mice were anesthetized with chloral hydrate 500 mg/kg, sacrificed and dissected for the different experiments. All efforts were made to minimize suffering. All animals have been raised and crossed in the indoor animal house in a 12 h light/dark cycle in a virus/antigen-free facility with controlled temperature and humidity and have been provided with water and food *ad libitum*.

### Generation of GFAP-IκkBaAA transgenic mice

The cDNA corresponding to mouse IkBαa (accession n° NM_010907) was PCR amplified from mouse brain mRNA and subcloned into the *Xho*I site of pSK-Bluescript II (Stratagene). The point mutations S32A and S36A were generated using the Quikchange site-directed mutagenesis kit (Stratagene). Rabbit b-globin poly-A signal (from nt 906 to nt 2560 of accession number K03256) was subcloned into the *Kpn*I site of pSK-I**κ**BαAA vector. A 2.2 kb fragment, corresponding to human GFAP promoter [22], [57], was subcloned into the pSK-I**κ**BαAA-polyA vector described above using a *Sal*I restriction site. The resulting GFAP-I**κ**BαAA expression cassette was gel purified, and transferred to the Transgenic Facility of EMBL (Monterotondo, Italy). DNA was microinjected into fertilized eggs of hybrid strains (e.g. B6CBA F1) and then introduced into pseudo-pregnant females. Transgenic offspring were identified originally by PCR, using the following oligos For 5′-ACTCCACTCCACTTGGCTGT-3′ and Rev 5′-CAAGTGCTCCACGATGGCCA-3′, and confirmed by Southern analyses.

Two GFAP-I**κ**BαAA transgenic mice lines (Tg-A and Tg-D) were backcrossed for six generations onto C57BL/6 background (98,4% C57BL/6 genetic background) before breeding with SOD1^G93A^ transgenic mice (strain B6.Cg-Tg(SOD1-G93A)-1Gur from The Jackson Laboratory, 99,99% C57BL/6 genetic background).

### Southern blot analysis and transgene copy number evaluation

10 µg of genomic DNA for each genotype, as well as the transgenic plasmidic construction, were digested with *Xho*I and DNA analized by standard Southern blot analysis. Considering that the aploid content of a mammalian genome is 3×10^9^ bp, that the transgenic mice are hemizygous and finally that GFAP-I**κ**BαAA trasnsgene is about 5×10^3^ bp, 10 copies of the transgene correspond to 0,1 ng of transgene DNA to 10 mg tail DNA [58].

### Western blot analysis

Protein samples (20 µg) were resolved on 12% SDS–polyacrylamide gel and transferred to Hybond-P membrane (Amersham). Membranes were blocked for 1 h in TBS, 0.1% Tween 20 and 5% non-fat dry milk, followed by an overnight incubation with primary antibodies (rabbit anti-COX2 Cayman, mouse anti-I**κ**Bα Calbiochem, mouse anti-p65 active subunit Millipore, mouse anti-GFAP Sigma-Aldrich, rabbit anti-TRL2 Cell Signaling, rabbit anti-TRL4 Cell Signaling, rabbit anti-IBA1 Wako) diluted in the same buffer. After washing with 0.1% Tween in Tris-buffered saline, the membrane was incubated with peroxidase-conjugated secondary antibody (Amersham) for 1 h, then washed and developed using the ECL chemiluminescent detection system (Roche). Densitometric analyses were performed using Image Quant T2 software program (GE Healthcare Life Science) and normalized against the signal obtained by reprobing the membranes with mouse anti-βactin (Sigma-Aldrich).

### Southern blot analysis

Genomic DNA from both transgenic lines and non transgenic mouse were digested with *XhoI* (BioLabs), run on 1% agarose gel and blotted on Hybond nylon membrane. Blots were incubated overnight with a labeled probe consisting of nucleotides 122–1066 of mouse IκBα gene (accession n° NM_010907), washed for 10 min at room temperature in 2×SSC and then for 20 min at 55°C in 0.5×SSC, 0.1% SDS. Gene copy number was evaluated by densitometric analysis with ImageQuant T2 software program (GE Healthcare Life Science) using endogenous IκBα gene as an internal standard.

### Electrophoretic mobility shift assay

Nuclear extracts were prepared and band-shift assays were performed as reported [17], using the oligonucleotide 5′-AGCTCAAACAGGGGGCTTTCCCT-3′ for NF-**κ**B site sequence (underlined sequence in fig. 2A).

### Primary astrocyte, microglial and neuron cultures

Primary astrocytes and microglia were prepared from 1- to 2-day-old mice using a modification of a published method [59]. Brains or spinal cords were dissected and meningeal tissue was stripped off. Brains were mechanically dissociated with fire-polished Pasteur pipettes and the resulting cell suspension was passed through a sterile nylon mesh (pore size, 70 mm; Falcon) in DMEM. After being washed by centrifugation at 200 g for 5 min, all cells from one brain were seeded into 25-cm^2^ culture flasks (Falcon) in DMEM supplemented with 20% FCS and antibiotics and were grown for 10–14 days at 37°C. In those conditions, neurons do not survive; microglial cells were collected by intensive shaking of culture flasks for 1 h at 37°C at 250 rpm in orbital shaker. Purified astrocytes and microglia were seeded on poly-L-lysine-coated coverslips and mouse recombinant TNFα (Sigma-Aldrich) (10 ng/ml) or LPS (Sigma-Aldrich, from *E. coli* strain 111:B4,) (10 µg/ml) was added to the cell culture medium for 20 min to 16 h. Cortical primary neurons were prepared from E16–E18 mouse embryo according to [60] and primary motor neurons according to [4].

### Immunofluorescence

Three mice per genotype at different disease stage (before onset, onset, symptomatic phase) were anesthetized with chloral hydrate (500 mg/kg) and perfused transcardially with 4% paraformaldeyde. The whole spinal cords were rapidly removed and postfixed overnight in 4% paraformaldeyde, cryoprotected in 20% sucrose and then stored at −80°C. Lumbar spinal cords were embedded in OCT freezing medium and 10 mm-thick sections were prepared with a cryostat. All sections were permeabilized with 0.1% Triton X-100 in PBS and non-specific binding was blocked with 10% goat serum, 2% bovine serum albumin, 0.1% Triton X-100 diluted in PBS for 1 h at room temperature. Sections were incubated with primary antibodies (mouse anti-GFAP diluted 1∶500, Sigma-Aldrich, rat anti-CD11b diluted 1∶200, Serotec) diluted in blocking solution, overnight at 4°C and then with secondary antibodies Cy3- and Alexa488-conjugated (Molecular Probe) diluted 1∶200 in blocking solution for 1 h at room temperature.

Primary cell cultures were grown on poly-L-lysine-coated glass slides, fixed in 4% paraformaldehyde for 10 min at 4°C and subsequently washed in PBS. Immunofluorescence analysis was performed as describe above. Rabbit anti-p65 (diluted 1∶200, Calbiochem), mouse anti-GFAP (diluted 1∶500, Sigma-Aldrich) and mouse anti-Smi32 (diluted 1∶500, Sternberg) were used as primary antibodies.

Sections and cells were analyzed with a confocal microscopy Leica TCS SP5 with LAS lite 170 image software.

### Quantification of microglia and astrocytes in the spinal cord sections

Images of the anterior horn area from every 10th lumbar spinal cord section (a total of 12 sections) from mice were photographed in the same conditions, followed by counting of CD11b-positive cells for activated microglia and GFAP-positive cells for astrocytes. The values for each sample were plotted and Pearson's correlation coefficient and significance of correlation were determined.

### RNA extraction and reverse transcription

Total RNA was extracted from mouse tissues treated with 3 mg/kg LPS (Sigma-Aldrich, from *E. coli* strain 111:B4) or saline and from astrocyte primary cultures treated with either 10 µg/ml LPS or 10 µg/ml recombinant TNFα Sigma-Aldrich), or saline at the selected time points using Trizol reagent (Invitrogen). The SuperScript™ III First-Strand reverse transcription system (Invitrogen) was used to synthesize cDNA, with 1 mg of total RNA and random hexamers, according to the manufacturer's instructions. Appropriate RT negative controls were included (without reverse transcriptase) to determine the presence of genomic DNA contamination. Samples with genomic contamination were treated with DNase I, Amp Grade (Invitrogen) following the manufacturer's instructions.

### Semi-quantitative RT-PCR

Primers were designed using Primer-3 software selecting a Tm around 54°C to allow amplification with the same cycling program. Primer sequences are: TNFα For 5′-CTGTGAAGGGAATGGGTGTT-3′, TNFα Rev 5′-CCCAGCATCTTGGTTTCTG-3′, IL-1β For 5′-CTCATTGTGGCTGTGGAGAA-3′, IL-1β Rev 5′-GCTGTCTAATGGGAACGTCA-3′, Fas For 5′-TATCAAGGAGGCCCATTTTGC-3′, Fas Rev 5′-TGTTTCCACTTCTAAACCATGCT-3′, iNOS For 5′-CATGCCATTGAGTTCATCAACC-3′, iNOS Rev 5′-TGTGAATTCCAGAGCCTGAAG-3′, β-actin For 5′-ATCCTGTGGCATCCATGAAAC-3′, β-actin Rev 5′-AACGCAGCTCAGTAACAGTC-3′. The number of cycles for amplification was determined empirically to allow quantification in the linear range of PCR. After reverse transcription, 1/10^th^ of the cDNA was used for each PCR reaction, except for β-actin where 1/30^th^ was used. PCR reactions were assembled with 0.2 µM of each primer, 2 mM dNTPs (Promega) and 2.5 U Go Taq (Promega). Cycling conditions were the same for all primer pairs: 94°C for 2 min, and then 30 cycles at 94°C for 30 s followed by 50°C for 45 s and 72°C for 45 s. PCR was carried out in GeneAmp PCR System2700 thermocycler (Applied Biosystems). 5 µl aliquots of the reaction mix were withdrawn at preestablished cycles, electrophoresed in 1% agarose gels, stained with ethidium bromide and analysed using VersaDoc Model 3000 (Biorad) coupled to Image Quant T2 software (GE Healthcare Life Science).

### Behavioural analysis

Behavioural analysis were performed according to the standard operating procedures indicated by [61]. All animals were tested twice a week for deficit in grip strength, Rotarod performance and body weight by the same operator who was blind to the genotype of mice. The progressive body weight loss was calculated as the difference from the maximum weight recorded for each animal. Analyses started at 30 days (progressive body weight) and 12 weeks (motor performances) of age. In the grip strength test, the time the mouse held on the inverted grid with both hind limbs was recorded. Each mouse was given up to three attempts to hold on to the inverted lid for a maximum of 90 s and the longest latency was recorded. Rotarod testing was performed using the accelerating Rotarod apparatus (Ugo Basile 7650 model). Time was started once the animals were positioned on the rotating bar, the rod was accelerated at a constant rate of 0.1 rpm/s from 3 rpm to 30 rpm for a maximum of 4 min 30″. The time (seconds) at which the animal fell from the bar was recorded. Three trials were given to each animal and the longest retention time was recorded. The onset of clear symptoms was considered when the mice showed the first impairment in grip strength. The symptomatic phase stage of disease was considered when the mice showed a 10% weight loss that was usually accompanied with the first impairment in Rotarod performance.

### Statistical analysis

Each experiment was repeated at least three times. Groups of at least three animals were used for biochemical analysis and unless indicated, all data are reported as mean+/−SEM. Behavioural analysis and survival data were analyzed with Kaplan–Meier curves and log rank test. Multiple group comparison was performed by one-way ANOVA with Bonferroni's post test and differences were declared statistically significant if p<0.05. All statistical computations were performed using GraphPad Prism 4.0 (GraphPad Software).
